# Sorting out inherent features of head-to-head gene pairs by evolutionary conservation

**DOI:** 10.1186/1471-2105-11-S11-S16

**Published:** 2010-12-14

**Authors:** Yun-Qin Chen, Hui Yu, Yi-Xue Li, Yuan-Yuan Li

**Affiliations:** 1 School of Life Science and Technology, Tongji University, Shanghai 200092, P.R. China; 2 Bioinformatics Center, Key Laboratory of Systems Biology, Shanghai Institutes for Biological Sciences, Chinese Academy of Sciences, Shanghai 200031, P.R. China; 3 Shanghai Center for Bioinformation Technology, Shanghai 200235, P.R. China

## Abstract

**Background:**

A ‘head-to-head’ (h2h) gene pair is defined as a genomic locus in which two adjacent genes are divergently transcribed from opposite strands of DNA. In our previous work, this gene organization was found to be ancient and conserved, which subjects functionally related genes to transcriptional co-regulation. However, some of the biological features of h2h pairs still need further clarification.

**Results:**

In this work, we assorted human h2h pairs into four sequentially inclusive sets of gradually incremental conservation, and examined whether those previously asserted features were conserved or sharpened in the more conserved h2h pair sets in order to identify the inherent features of the h2h gene organization. The features of TSS distance, expression correlation within h2h pairs and among h2h genes, transcription factor association and functional similarities of h2h genes were examined. Our conservation-based analyses found that the bi-directional promoters of h2h gene pairs are most likely shorter than 100 bp; h2h gene pairs generally have only significant positive expression correlation but not negative correlation, and remarkably high positive expression correlations exist among h2h genes, as well as between h2h pairs observed in our previous study; h2h paired genes tend to share transcription factors. In addition, expression correlation of h2h pairs is positively related with the TF-sharing and functional coordination, while not related with TSS distance.

**Conclusions:**

Our findings remove the uncertainties of h2h genes about TSS distance, expression correlation and functional coordination, which provide insights into the study on the molecular mechanisms and functional consequences of the transcriptional regulation based on this special gene organization.

## Background

A ‘head-to-head’ (h2h) gene pair is defined as a genomic locus in which two adjacent genes are divergently transcribed from opposite strands of DNA, and, the region between the two transcription start sites (TSSs), commonly shorter than 1000 bp, is termed the ‘bi-directional promoter’ [1,2]. H2h gene pairs have been found to be a unique gene arrangement in vertebrates, particularly in human genome [2,3]. Recent studies have been characterizing the sequential features of the bi-directional promoters [4,5], exploring the co-regulation pattern among h2h gene pairs [6], and investigating their functional relevance such as that with tumorigenesis [6,7]. Taken together, these findings seem to echo a preliminary conclusion we made in 2006 [3]: “the head-to-head gene organization is ancient and conserved, which subjects functionally related genes to correlated transcriptional regulation and thus provides an exquisite mechanism of transcriptional regulation based on gene organization.” However, there is still some doubt or uncertainty on specific features of h2h gene pairs to be resolved by close-up investigations. For instance, we observed in our previous study that pairs with TSSs separated 1- to 400- bp apart formed the peak columns in the TSS distance distribution, and we anticipated a compression of these columns to a narrower or sharper region. Although we did witness a significant inflation of rat h2h pairs in the 1- to 400- bp TSS distance group during a three-year update, we still could not affirm how long a bi-directional promoter most optimally is. For another example, we observed positive, negative, and alternative expression correlation between h2h paired genes, but negative correlation was not confirmed by peer studies [2,4], and a novel opinion came up that significant expression correlation may exist among h2h genes (not necessarily within pairs) [4]. Other aspects of h2h gene pairs, such as their transcriptional regulation and function coordination, are still ambiguous to some extent.

In the present study, we sorted previously asserted features of h2h gene pairs, trying to remove these uncertainties and identify the inherent features of this gene arrangement. Based on a commonly accepted principle that evolutionarily conserved facts are by all means associated with biological significances [8], we believed that the more conserved head to head gene pairs, of greater biological importance, must more likely represent the inherent features of h2h gene pairs. Therefore, we assorted human h2h pairs into four sets of incremental conservation in vertebrates, and sorted out inherent features of vertebrate h2h gene pairs by comparing the four h2h pair sets on a series of points. We gave comprehensive analyses on h2h pair features including TSS distance, expression correlation nature, transcription factor association, and functional coordination, and provided unambiguous judgment on specific features according to their evolutionary conservation. This study provides useful clues for the mechanism study on the transcriptional regulation of the h2h gene organization.

## Methods

### Data sources

According to DBH2H [9](http://lifecenter.sgst.cn/h2h/), we determined human, chicken, and *fugu* H2h gene pairs, and the TSS Distances of each pair. Expression correlation data were downloaded from two sources: DBH2H [9] (http://lifecenter.sgst.cn/h2h/) and COXPRESdb [10](http://coxpresdb.jp/).

Transcription factor association of h2h gene pairs was enabled by the integrated transcription factor platform [11] (ITFP, http://itfp.biosino.org/itfp/), which maintains both experimentally verified TFs and *in-silico* predicted TFs.

Annotation of Gene Ontology (http://www.geneontology.org) terms of h2h genes was aided by Bioconductor packages org.Hs.eg.db 2.3.6 and GO.db 2.3.5.

### Expression correlation of head-to-head gene pairs

From DBH2H, we got Pearson and Spearman expression correlation data of human h2h gene pairs on 43 public datasets respectively; from COXPRESdb, we got the Pearson expression correlation value, as well as a relative correlation index MR (Mutual Rank) [12], for each of all possible pairs among 19777 human genes. COXPRESdb data were calculated from gene expression profiles across 3749 human samples.

Specifically, MR is defined as the geometric mean of the reciprocal relative expression correlation ranks with respect to the two genes of a pair:  (A and B stand for two genes).Additionally, we calculated another relative expression correlation index RR (Relative Rank), defined as RR(A,B)=min(Rank(A->B),Rank(B->A)). Wherever one single expression correlation value was used for summarizing an h2h pair set, we performed the average operation over all COXPRESdb values of the set. A total of 1447000 (1447*1000) of random gene pairs and 5252 same-strand adjacent pairs involving 2835 h2h genes were determined for control. Their expression correlation values were also taken from the COXPRESdb data.

With DBH2H expression correlation data, we determined for each h2h pair the significant correlations with the corresponding p-values lower than 0.05. As the significant correlations could be positive or negative, we got three total numbers respectively: SP, SN, and SP+SN. Dividing the three total numbers with the number of investigated datasets separately, we obtained the SPR (Significant Positive Ratio), SNR (Significant Negative Ratio), and SR (Significant Ratio), representing the proportion of significant positive correlation, significant negative correlation, and significant correlation of an h2h pair, respectively. Note that SPR+SNR=SR. When different sets of h2h pairs were compared in terms of expression correlation level, we reported the average SPR, SNR, or SR of each set.

### Functional similarities between head-to-head paired genes

The Gene Ontology (GO) [13] annotation system was used to annotate h2h genes. In GO system, a gene can be annotated to more than one functional term, and it is common to see one gene annotated simultaneously in three GO subsystems. When both genes are annotated in a same GO category, we judged that this gene pair was annotated by the GO category. The Lin semantic measure [14], derived from Resnik’s GO term similarity measure [15], is a normalized index ranging between 0 and 1. Resnik’s similarity measures relies on the notion of the so-called minimum subsumer *t* of two GO terms *t1* and *t2*, which is the lowest common ancestor in the GO hierarchy. Its information content IC_ms_, which is the Resnik semantic similarity measure between *t1* and *t2*, is given by Equation 1. Here *Pa(t1, t2)* denotes the set of all common (also indirect) ancestors shared by GO terms *t1* and *t2*, and *IC(t)* is defined as the negative logarithm of the probability of observing term t (p(t)). *P(t)* can be technically approximated by the number of genes annotated to term *t*. Finally, the Lin semantic similarity measure is determined through normalizing the Resnik measure to the range between 0 to 1 (Equation 2).(1)(2)

The calculations of functional similarity were performed using the GOSim [16] package, version 1.2.1.1(http://cran.r-project.org/web/packages/GOSim/index.html) in the R environment (http://www.r-project.org/) . We also calculated the functional similarity of random pair sets with the same size of annotated h2h gene pairs, with iteration 100 times.

## Results and discussion

We studied head-to-head gene organization in vertebrates by selecting *fugu rubripes*, *gallus gallus, mus musculus,* and *homo sapiens* genomes as the representative vertebrate phylogeny. *Fugu* has the shortest known genome (~365 Mb) of any vertebrate species - around one eighth of the size of the human genome [17], therefore roughly representing the start-point of the vertebrate phylogeny. The chicken has a genome of 1.2 Gb, approximately 40% of the size of the human genome, and is the premier non-mammalian vertebrate model organism [18]. Mouse and human are two of the most well-studied mammalian model animals, and, in contrast to *fugu*, they approximately represent the end-point of the vertebrate phylogeny. Based on data downloaded from DBH2H [9], 1447 human h2h gene pairs were assorted into four sequentially inclusive sets: set H, including all 1447 human pairs; set HM, including 191 pairs conserved between human and mouse; set HMC, including 77 pairs conserved across human, mouse and chicken; set HMCF, including the 14 pairs conserved across human, mouse, chicken and *fugu.* The four sets of human h2h pairs with gradually increasing conservation levels were compared in terms of genomic TSS distance, expression correlation, transcriptional factor association, and functional similarity. In each analysis, we firstly compared the feature of the largest set H and that of a randomly sampled gene pair set or a set of ‘adjacent’ gene pairs composed of h2h genes and their adjacent genes. If a statistically significant difference between set H and the random set (or the adjacent set) was observed, we furthermore compared the feature between the four h2h pair sets, and relied on two-group t-tests or wilcoxon rank-sum tests to decide whether there was statistically significant difference between the different conservation levels. If a feature was validated in both stages of statistical tests, we declared it was an inherent feature of the h2h gene organization; if a feature was not validated by either stage, or if it showed contrary trend in the conservation-based test, we tentatively negated it. If a feature had significant difference between set H and the random set (or the adjacent set), but did not display significant difference, in consistent directions, between the different conservation levels, we postponed the related declaration to future studies where hopefully expanded data would lead to an unambiguous conclusion

### H2h pair with 1-100 TSS distance probably has a functional bi-directional promoter

**Figure 1:**
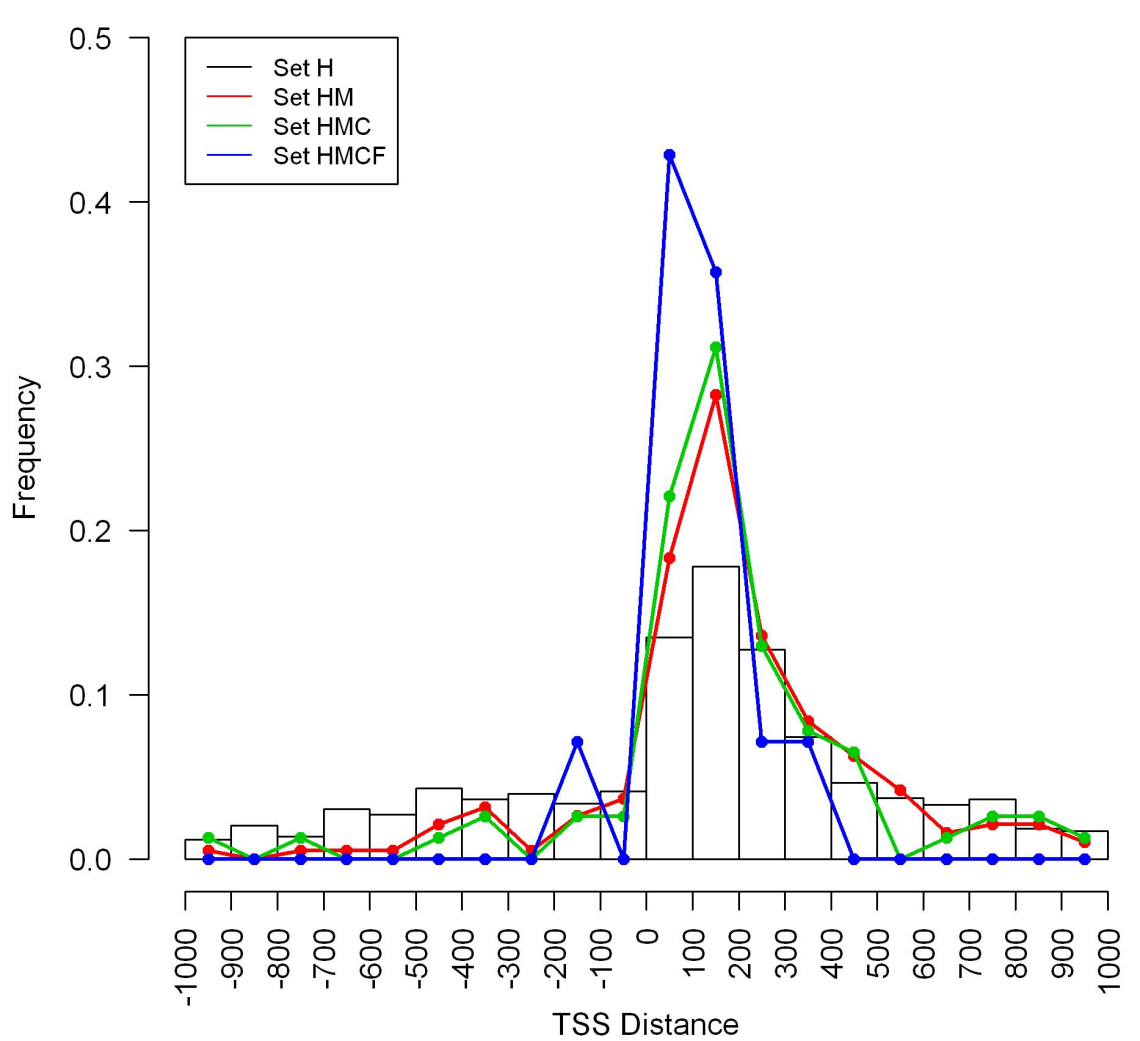
TSS distance distributions of four h2h pair sets

Given the TSS distances of all h2h pairs in hand, we examined the TSS distance distributions of the four h2h pair sets in a comparative manner. We found that non-overlapping h2h pairs separated by less than 400 bp formed the majority of the four sets (Table 1), resonating the earlier observation of human, mouse, and rat h2h pairs [3]. Remarkably, the peak TSS distance interval in HMCF was (0, 100], different from the counterpart peaks (100, 200] in H, HM, and HMC (Table 1 and Figure 1), and a gradually incremental trend of the proportion of the four h2h gene pairs (from set H to set HMCF) located in (0, 100] was statistically significant (chi-squared test, p=0.001). This result accorded with our previous guess that ‘the peak column (101 to 200 bp) in the TSS distance distribution might actually move somewhat to the left or be much sharper’ [3].

**Table 1:** Percentages of h2h pairs within particular TSS distance intervals

	(0, 100) bp	(100, 200) bp	(0, 400) bp
Set H	14.2%	20%*	54.9%
Set HM	18.3%	28.3%*	68.6%
Set HMCF	22.1%	31.2%*	74.0%
Set HMCF	42.9%*	35.7%	92.9%

*The largest percentage within a 100-bp interval. It can be seen that the peak intervals at the four gradually-increasing conservation levels are (100, 200) bp, (100, 200) bp, (100, 200) bp, and (0, 100) bp, respectively.

Considering another fact that the core promoter [19], or the minimal portion of the promoter required to properly initiate transcription, is confined to 100 bp region upstream of a TSS, we have increased confidence in that the h2h pair with their TSSs separated 1-100 bp most likely has a functional bi-directional promoter, which has biological relevance to the co-regulation of the two genes. As we witnessed a compression of TSS distances of rat h2h pairs between two batches of analyses [3,9], we anticipated an impending replacement of the then peak column (100, 200] by (0, 100] in future data updates.

We also related TSS distance with expression correlation of the h2h paired genes, but found no significant relationship between them, no matter in set H or in the more conserved set HM, HMC and set HMCF. Even if we studied the overlapping and non-overlapping h2h pairs separately, we still did not detect any correlation between TSS distance and expression correlation. Hence, we stuck to our postulation that a bi-directional promoter tend to coordinately regulate the transcriptions of h2h paired genes in a TSS distance-unrelated manner [3].

### Significant positive expression correlation within h2h pairs and among h2h genes

**Figure 2:**
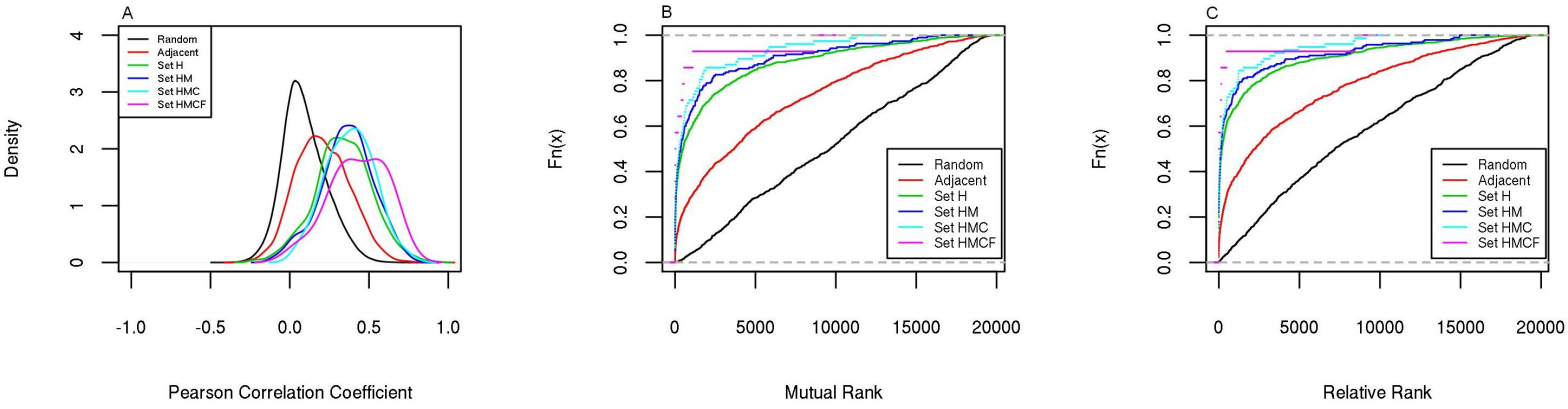
**Distribution of Expression correlation indices for gene pairs from five different sets**. Indices used: Pearson’s Correlation Coefficient (A), Mutual Rank (B), and Relative Rank (C). Data were from COXPRESdb.

Based on the expression correlation data obtained from COXPRESdb [10], we compared the expression correlation level among the four h2h pair sets. There measures, Pearson Correlation Coefficient (PCC), Mutual Rank (MR) and Relative Rank (RR), were used to evaluate gene coexpression level (see Methods). It was found that the PCCs within h2h gene pairs were significantly higher than those of random pairs (two-group t-test p<0.05), and higher than same-strand adjacent pairs involving h2h genes too (two-group t-test p<0.05); the similar results were observed for MR and RR as well (two-group t-test p<0.05 for h2h vs. random and h2h vs. adjacent comparisons). Furthermore, the PCCs and MRs were increasing and decreasing, respectively, with the conservation level. These comparisons were visualized in Figure 2.

The coexpression strengthening with conservation level was also revealed in DBH2H data (Table 2). As DBH2H [9] provides dataset-specific expression correlation values of h2h pairs, we were able to know the Significance Ratio (SR) of each h2h pair, which represented the fraction of datasets in which an h2h pair was significantly correlated (p<0.05 for a specific correlation coefficient). As revealed in Table 2, one notable fact that on average an h2h pair of set H had a SR of around 50% (Pearson correlation coefficient) indicated a remarkable tendency of transcription co-regulation between h2h paired genes, and this tendency was further supported by the other fact that the SR statistic was increasing with the conservation level (Table 2). The trend was similar for Pearson and Spearman correlation coefficients (Table 2). Taking together the results from COXPRESdb and DBH2H, we approved a significant tendency of transcription coordination between a pair of h2h genes. In addition, as the median RRs of h2h gene pairs were quite small (4.5 for HMCF, 102 for HMC, 137.5 for HM and 190 for H), we infer that the strongest correlation associated to an h2h gene probably exists between this gene and its h2h pairing partner.

**Table 2:** Expression correlation within h2h pairs in DBH2H

	Correlation		Positive Correlation		Negative Correlation	
	
	Pearson^a^	Spearman^b^	Pearson	Spearman	Pearson	Spearman
Set H	0.55	0.51	0.61	0.54	0.42	0.42
	(52.2%)^c^	(43.2%)	(45.3%)	(36.2%)	(6.9%)	(7.0%)
Set HM	0.57	0.52	0.62	0.55	0.44	0.44
	(55.9%)	(47.3%)	(48.6%)	(40%)	(7.2%)	(7.3%)
Set HMC	0.57	0.53	0.62	0.55	0.46	0.46
	(55.8%)	(47.6%)	(48.3%)	(40%)	(7.4%)	(8.0%)
Set HMCF	0.59	0.55	0.64	0.59	0.45	0.43
	(57.5%)	(50.5%)	(51.3%)	(44%)	(6.2%)	(6.5%)

^a^ Two-group *Wilcoxon* test, p<0.05 for set H vs set HM

^b^ Two-group *Wilcoxon* test, p<0.05 for set H vs set HM

^c^ A percentage in round brackets refers to the average Significance Ratio (SR).

Furthermore, we examined whether negative correlation is an inherent feature of h2h gene pairs. We first noticed that, in COXPRESdb, set H had a smaller fraction of gene pairs with negative expression correlation than random pair set and adjacent pair set (chi-squared test, p<0.01), and the fractions in sets HM, HMC and HMCF were even smaller (0.02 in HM, 0 in both HMC and HMCF). Additionally, the average correlation values separately for positive and negative correlation of each h2h pair were examined according to DBH2H [9]. Interestingly, we observed a stable increment in positive correlation between the four h2h sets, but no similar trend in negative correlation. Moreover, we discerned a remarkable preponderance of positive correlation over negative correlation, as the Significance Ratios (SRs) were mostly contributed by Significant Positive Ratios (SPRs) (Table 2). The average ‘Significant Negative Ratio’ (SNR) of h2h pairs, at any conservation level, was lower than 10%, and it even decreased a little from set H to set HMCF (Table 2). A more typical decreasing trend was found with the average proportion of datasets showing negative correlation (data not shown). This indicated that negative correlation was quite likely not an inherent feature of the h2h gene arrangement, in accordance with a previous claim that there was no evidence for negative expression correlation of a significant number of gene pairs [20].

**Figure 3:**
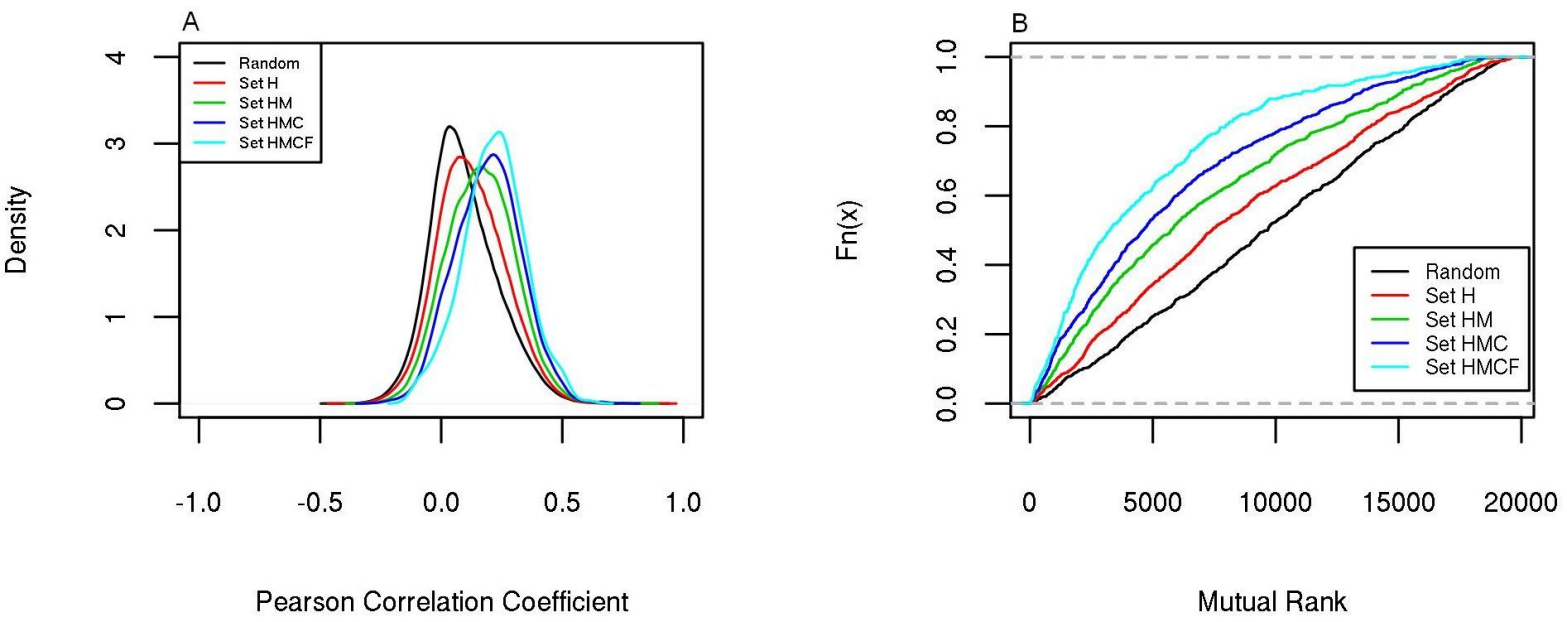
**Distribution of Expression correlation indices for possible pairs formed by genes from four different sets.** Indices used: Pearson Correlation Coefficient (A), Mutual Rank (B). Data were from COXPRESdb.

It was proposed that expression correlation may happen not only within h2h gene pairs, but also across different h2h pairs [21]. To verify this hypothesis, we determined all possible gene pairs within the scope of h2h genes while purposely excluded the actual h2h pairs, and checked their average COXPRESdb PCC and the average MR at the four conservation levels. We saw that PCC was steadily increasing while MR was steadily decreasing with the conservation level (p<0.001, *Wilcoxon* tests), and that both statistics in all four h2h sets were significantly different from the counterpart statistics of random gene pairs (Figure 3, Wilcoxon test, p<0.01). Moreover, we inspected expression correlation between h2h genes and the other genes that are not involved in the h2h arrangement. It was found that the expression correlation at all conservation levels were very close to random gene pairs (data not shown).

In summary, our conservation-based analyses validated the significant positive coexpression tendency within and between h2h gene pairs, but negated the universal existence of negative expression correlation of h2h pairs. The intra-pair expression correlation level seems higher than the inter-pair one. A further study on the roles of h2h genes in coexpression networks is still going on.

### High expression correlation owes to shared transcription factors

Despite the consensus that h2h gene pairs are often co-transcribed, the transcriptional regulation mechanisms of h2h gene pairs remain unclear. Lin et al [4] addressed this issue by discriminating over-represented and under-represented transcription factor binding sites (TFBSs) from bi-directional promoters. We wanted to complement their work by emphasizing the transcription factors (TFs) which potentially regulate h2h genes.

We tried associating TFs to human h2h genes (within set H) based on the experiment and computation-based ITFP database [11] and the experiment-based TRANSFAC database. Through ITFP, we determined 207 ‘TF-associated h2h gene pairs’ of which the two h2h paired genes were both associated to TFs; this number was by far larger than that obtained through TRANSFAC. By adopting ITFP, therefore, we achieved an optimal trade-off between data size and credibility.

**Figure 4:**
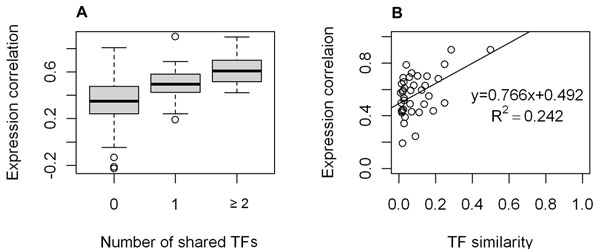
**TF similarity is positively related with expression correlation of h2h gene pairs**. (A) boxplots of expression correlation values of four h2h pair groups with different level of TF similarities. Pairs sharing at least one TF ( 2^nd^ and 3^rd^ ) boxplots displayed a significantly higher expression correlation than those with no common TF (1^st^ boxplot): mean values 0.564 vs. 0.355, two group t-test p =1.21e-09; pairs sharing two or more TFs (3^rd^ boxplot) displayed a significantly higher expression correlation than those with only one common TF (2^nd^ boxplot): mean values 0.619 vs 0.496., two group t-test p=0.02.(B) the scatter plot of expression correlation values vs. TF similarities (defined as the fraction of shared TFs in the union TFs).

Of these 207 TF-associated pairs, 168 shared no common TF, 18 shared one common TF, and 21 shared more than one common TFs (Table 3). Comparing the expression correlations among the four groups of TF-associated pairs, we found that the groups with more common TFs consistently displayed higher expression correlation (Figure 4A). As a matter of fact, we observed a positive correlation between the expression correlations and TF similarities of the 39 TF-sharing h2h pairs, provided that TF similarity was defined as the fraction of shared TFs in the union TFs (Figure 4B). It was noted that the proportion of TF-sharing pairs within TF-associated pairs, 18.8%, was statistically higher than 12.4%, the counterpart statistics from same-strand adjacent pairs at p<0.05. Projecting the 207 TF-associated h2h gene pairs into the three conserved h2h pair sets HM, HMC and HMCF, we obtained 64 in set HM, 30 in set HMC, and 5 in set HMCF (Table 3). As Table 3 showed, the TF-sharing pairs accounted for an increasing fraction of the TF-associated pairs as the conservation level increased. In sets HM and HMC, we also noted a possible positive relationship between expression correlation and TF-sharing, although the p-values were not significant, possibly due to the minimal sample sizes (data not shown).

**Table 3:** TF-association of h2h pairs

	TF-sharing pairs	TF-exclusive pairs	Proportion of TF-sharing pairs in annotated pairs
Adjacent	53	374	12.4%
Set H	39	168	18.8%
Set HM	13	51	20.3%
Set HMC	6	24	20%
Set HMCF	3	2	60%

In addition, there were seven h2h pairs in which one gene was a TF regulating the other one (Table 4). These self-regulating h2h pairs demonstrated rather high expression correlation, even higher than the group of 39 TF-sharing h2h pairs (two group t-test p=0.0004).

**Table 4:** Seven h2h gene pairs in which one gene regulates the other

	Pearson Correlation Coefficient (PCC)	Mutual Rank (MR)
CSTF1 -> AURKA	0.55	68.2
DTX3L -> PARP9	0.807	1
WDSOF1 -> SLC25A32	0.701	1.7
MCM4 -> PRKDC	0.7	7.8
RECQL -> GOLT1B	0.627	1.4
NUFIP1 -> KIAA1704	0.487	29.9
POLR3K -> C16orf33	0.701	1

**Table 5:** Functional similarities of h2h gene pairs

	BP	MF	CC
Adjacent gene pairs	0.29 (1603) ^a^	0.34 ^b^ (1859)	0.44 ^b^(1993)
Set H	0.33^c^ (405)	0.37 ^c^(492)	0.51 ^c^ (508)
Set HM	0.29 (105)	0.36 (112)	0.51 (123)
Set HMC	0.21(48 )	0.33 (42)	0.45 (53)
Set HMCF	0.38 (8)	0.27 (7)	0.59 (9)

^a^ Number of annotated h2h pairs are shown in round brackets.

^b^ Functional similarities of set H were significantly higher than h2h genes’ adjacent gene pairs (p<0.05).

^c^ Functional similarities of set H, at any GO subsystem, were significantly higher than random pair sets of comparable sizes (p<0.01).

**Table 6:** Average functional similarity of h2h gene pairs delimited by expression correlation thresholds

	Functional Similarity		
	
	PCC>0.355*	PCC>0.5	PCC>0.6
Set H	0.576	0.594	0.654
Set HM	0.538	0.532	0.564
Set HMC	0.512	0.541	0.693
Set HMCF	0.687	0.567	0.848

* 0.355 was the median expression correlation of GO-annotated human h2h gene pairs.

According to our results, h2h paired genes tend to share TFs, and the TF sharing degree is positively correlated with expression correlation. Sharing regulators seems to be a universal characteristic of h2h gene pairs which partially explains the significant positive expression correlation between h2h paired genes.

### Functional similarity analysis of h2h gene pairs

Based on gene function classification system Gene Ontology (GO) [13]), we determined GO-annotated h2h pairs for the three subsystems of GO respectively (“Biological Process” or BP, “Molecular Function” or MF, and “Cellular Component” or CC), and these annotated pairs were projected in all four h2h sets of different levels of conservation (Table 5). Note that an annotated h2h pair is one having its both genes annotated in a common GO subsystem, and the functional similarity (semantic similarity) of each pair of h2h genes is measured using the method proposed by Lin [14]. Since each pair of genes was tagged with three semantic similarities calculated in the three GO subsystems separately, the maximum semantic similarity of the three was taken as the representative functional similarity of an h2h pair. Firstly, we found out that, in any GO subsystem, human h2h gene pairs (Set H) manifested significantly higher functional similarity than random pairs (Wilcoxon test, P<0.01); the same conclusion was drawn when compared to same-strand adjacent pairs in subsystems CC and BP (Wilcoxon test, p<0.05). And the average functional similarities in BP and CC in set HMCF were higher than those in set H (Table 5). However, functional similarity in MF dropped with the conservation level (Table 5). Secondly, we noted a significant correlation between functional similarity and expression correlation of human h2h paired genes (R=0.177, p-value= 1.044e-05). As Table 6 showed, h2h gene pairs with higher expression correlation were associated with higher functional similarity, and this phenomenon was conserved in sets HM, HMC and HMCF. We particularly pointed out that the correlation between functional similarity and negative expression correlation degenerated (R=0.01, see Additional file 1), which was possibly another evidence negating the negative expression correlation of h2h gene pairs.

Taking the above two points together, there seems to be a functional similarity between h2h organized genes and a correlation between the functional coordination and the expression correlation. In all, through sharing bi-directional promoters, h2h gene pairs tend to be coexpressed and their products tend to perform similar functions. As we previously proposed, similar to operons in bacteria, h2h gene arrangement is an economic and ingenious strategy in eukaryotes to achieve coordination between functionally related genes.

## Conclusions

In this work, using recently accumulated genomic and expression data, we systematically re-examined the diverse features of head-to-head gene pairs previously proposed [3] and verified the features inherent in the h2h gene arrangement based on the evolutionary conservation. On a whole, most discoveries or hypotheses made in the previous work were confirmed: the functional bi-directional promoters of h2h gene pairs are most likely shorter than 100 bp; h2h paired genes show significantly high positive expression correlation; h2h paired genes are involved in related functions and the functional similarity is positively correlated with gene pair expression correlation. However, negative expression correlation is probably not an inherent feature of h2h gene pairs. As an additional discovery, we found that the expression correlation among all h2h genes (not necessarily forming h2h pairs) are higher than the background level, indicating that h2h genes in aggregate may subject to shared regulatory program. We further demonstrated that each h2h gene pair statistically tends to share common transcription factors, which in part explains the unusually high expression correlation among h2h genes.

Our present findings resolved the uncertainties on TSS distance, expression correlation nature, and functional coordination of h2h gene pairs, which may benefit future studies on the transcriptional regulation mechanism and the biological significance of h2h gene pairs.

## Competing interests

The authors declare that they have no competing interests.

## Authors' contributions

YYL designed the study. YQC and HY performed the analyses. YQC, YYL and HY analyzed the results. HY, YQC and YYL drafted the manuscript. YYL and YXL supervised the study.

## Supplementary Material

Additional file 1 Functional similarity of negatively correlated gene pairs
The Supplementary Table 1 contains the functional similarity of negatively correlated h2h gene pairs. The PCC between “Functional similarity” and “PCC” were merely 0.01. Click here for file
