# Supplementary material for: Sorting out inherent features of head-to-head gene pairs by evolutionary conservation
Source: BMC Bioinformatics. 2010 Dec 14;11(Suppl 11):S16. doi: 10.1186/1471-2105-11-S11-S16 (PMC3024869; doi:10.1186/1471-2105-11-S11-S16)
Supplement: Additional file 1 — Functional similarity of negatively correlated gene pairs The Supplementary Table 1 contains the functional similarity of negatively correlated h2h gene pairs. The PCC between “Functional similarity” and “PCC” were merely 0.01. [file 1471-2105-11-S11-S16-S1.pdf]

Supplementary Table 1. Functional similarity of negatively correlated h2h gene pairs. The PCC between "Functional similarity" and "PCC" were merely 0.01.

PosGeneID	NegGeneID	PosSymbol	NegSymbol	Functional similarity	MR	PCC
149465	10969	WDR65	EBNA1BP2	0.061	16981.1	-0.115
163049	57474	ZNF791	ZNF490	1	15814.7	-0.065
169436	6836	C9orf96	SURF4	0.157	14887	-0.021
27158	94107	NDOR1	TMEM203	0.072	17612.1	-0.133
2967	1967	GTF2H3	EIF2B1	0.510	15692.1	-0.075
388531	84079	RGS9BP	ANKRD27	0.072	16328	-0.097
4720	9507	NDUFS2	ADAMTS4	0.522	17881.3	-0.12
54814	6633	QPCTL	SNRPD2	1	16365.3	-0.084
57731	645	SPTBN4	BLVRB	1	14677.3	-0.023
80128	200185	TRIM46	KRTCAP2	0.134	16148.5	-0.063
8799	114814	PEX11B	GNRHR2	0.863	18990.2	-0.226
